# Multiple effects of toxins isolated from *Crotalus durissus terrificus* on the hepatitis C virus life cycle

**DOI:** 10.1371/journal.pone.0187857

**Published:** 2017-11-15

**Authors:** Jacqueline Farinha Shimizu, Carina Machado Pereira, Cintia Bittar, Mariana Nogueira Batista, Guilherme Rodrigues Fernandes Campos, Suely da Silva, Adélia Cristina Oliveira Cintra, Carsten Zothner, Mark Harris, Suely Vilela Sampaio, Victor Hugo Aquino, Paula Rahal, Ana Carolina Gomes Jardim

**Affiliations:** 1 Genomics Study Laboratory, São Paulo State University, IBILCE, S. José do Rio Preto, São Paulo, Brazil; 2 Laboratory of Virology, School of Pharmaceutical Sciences of Ribeirão Preto, University of São Paulo, São Paulo, Brazil; 3 Laboratory of Toxinology, School of Pharmaceutical Sciences of Ribeirão Preto, University of São Paulo, São Paulo, Brazil; 4 School of Molecular and Cellular Biology, Faculty of Biological Sciences, and Astbury Centre for Structural Molecular Biology, University of Leeds, Leeds, United Kingdom; University of Cincinnati College of Medicine, UNITED STATES

## Abstract

*Hepatitis C virus* (HCV) is one of the main causes of liver disease and transplantation worldwide. Current therapy is expensive, presents additional side effects and viral resistance has been described. Therefore, studies for developing more efficient antivirals against HCV are needed. Compounds isolated from animal venoms have shown antiviral activity against some viruses such as *Dengue virus*, *Yellow fever virus* and *Measles virus*. In this study, we evaluated the effect of the complex crotoxin (CX) and its subunits crotapotin (CP) and phospholipase A_2_ (PLA_2_-CB) isolated from the venom of *Crotalus durissus terrificus* on HCV life cycle. Huh 7.5 cells were infected with HCVcc JFH-1 strain in the presence or absence of these toxins and virus was titrated by focus formation units assay or by qPCR. Toxins were added to the cells at different time points depending on the stage of virus life cycle to be evaluated. The results showed that treatment with PLA_2_-CB inhibited HCV entry and replication but no effect on HCV release was observed. CX reduced virus entry and release but not replication. By treating cells with CP, an antiviral effect was observed on HCV release, the only stage inhibited by this compound. Our data demonstrated the multiple antiviral effects of toxins from animal venoms on HCV life cycle.

## Introduction

Hepatitis C is a disease caused by Hepatitis C virus (HCV) infection, essentially characterized by liver inflammation. Chronic infection may progress to cirrhosis or hepatocellular carcinoma and represents one of the major causes of liver diseases and transplants [1]. Approximately 130–150 million people are chronically infected worldwide [2].

HCV is grouped into the genus *Hepacivirus* within the family Flaviviridae. Virions are enveloped and present and a single stranded positive-sense RNA genome surrounded by a proteic capsid [3]. There is no vaccine for preventing HCV infection and for many years the interferon-based was the only treatment against HCV infection [4].

Recently, the addition of the direct-acting antiviral agents (DAAs) which target viral proteins such as NS5A and NS3-4A to the standard interferon therapy or the interferon-free regimens increased the sustained virological response (SVR) rates [5,6]. Sofosbuvir and daclatasvir are two oral DAAs which increase SVR even for difficult-to-treat genotypes, demonstrating high tolerance for patients. However, DAAs based therapies cost approximately US$84,000 for 12-week treatment, making this regimens inaccessible for many countries [7,8]. Additionally, studies have demonstrated that specific mutations may confer viral resistance to its treatment [9]. Therefore, the search for new therapeutics for the treatment of HCV infection is of great interest and could provide a substantial benefit to the global public health [10].

In this context, compounds extracted from natural sources have shown therapeutic potential for treating chronic hepatitis C [11–13]. Toxins isolated from animals as the poisonous snakes have been widely studied with respect to their applications, including antiviral properties [14–17]. Snake venoms are a mixture of bioactive compounds that possess numerous metabolic activities [18]. These compounds previously demonstrated to inhibit the life cycle of a range of viruses, including the *Flaviviridae* family. Components of snake venoms have shown antiviral activity against *Dengue virus* (DENV), *Yellow Fever virus* (YFV), *Oropouche virus* (OROV), *Mayaro virus* (MAYV) [16,19], *Measles virus* (MeV) [17] and *Human immunodeficiency virus* (HIV) [15,20]. Therefore, toxins isolated from venomous snakes may provide an alternative approach for the development of new antivirals.

In this study, we investigated the antiviral effects of the complex crotoxin and its subunits crotapotin and phospolipase A_2_ isolated from the venom of *Crotalus durissus terrificus* [19,21] on HCV life cycle. The data obtained showed that these toxins can inhibit different stages of the viral replicative cycle.

## Material and methods

### Toxins

The crude venom from *Crotalus durissus terrificus* was purchased from the serpentarium "Animal Toxin Extraction Center" (CETA) duly registered in Ministry of the Environment, nr. 3002678. The extraction was performed by Jairo Marques do Vale (CETA). The venom was collected from 28 specimens (pool) from Morungaba—SP collection.

**Fig 1:**
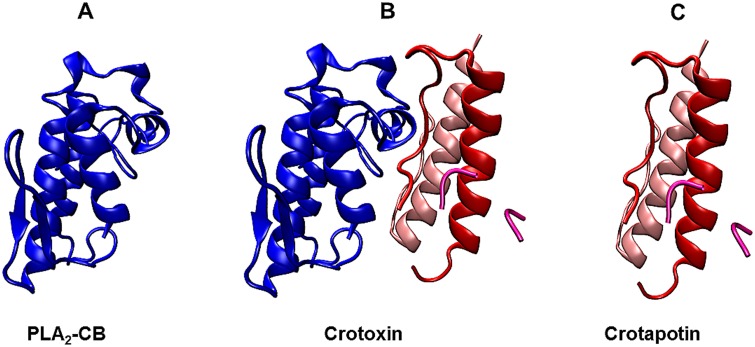
Crystal structure of the complex crotoxin from *Crotalus durissus terrificus* venom. The basic subunit (PLA_2_-CB) is displayed in blue (A). The overall structure of crotoxin complex (B). The three chains of acid subunit (crotapotin) is shown in red [α], light pink [β] and pink [γ] and (C) (PDB ID: 3R0L).

Isolation and purification of the crotoxin complex (CX), and its subunits phospholipase A2 (PLA2-CB) and crotapotin (CP) (Fig 1) were carried out at the Laboratory of Toxinology of the School of Pharmaceutical Sciences of Ribeirão Preto, University of São Paulo (IBAMA authorization: 1/35/1998/000846-1), under the supervision of Prof Suely Vilela Sampaio, as previously described in details [19,21].

Lyophilized toxins were dissolved in PBS (Phosphate-Buffered Saline), filtered and stored at -80°C. Compounds were diluted in complete medium immediately prior to the experiments.

### Cell culture

The human hepatoma cell line Huh-7.5 [22] was grown in Dulbecco’s modified Eagle’s medium (DMEM; Sigma–Aldrich, USA) supplemented with 100 U/mL penicillin (Gibco Life Technologies, USA), 100 mg/mL streptomycin (Gibco Life Technologies, USA), 1% non-essential amino acids (Gibco Life Technologies, USA), 1% HEPES (Gibco Life Technologies, USA) and 10% fetal bovine serum (FBS; Cultilab, BR) at 37°C in a humidified 5% CO_2_ incubator. The subgenomic replicon (SGR) cell lines harboring genotype 2a SGR-Feo-JFH-1 [23] or genotype 1b SGR BM45-Feo [24] was maintained in DMEM supplemented with 500 μg/mL G418 (Sigma-Aldrich, USA).

### Cytotoxicity assay

Cytotoxicity of toxins was measured by the MTT [3-(4,5-dimethylthiazol-2-yl)-2,5-diphenyl tetrazolium bromide] (Sigma–Aldrich, USA) method [25]. Huh-7.5 or SGR-harboring cells were cultured in DMEM medium in a 96-well plate at a density of 5 x 10^3^ per well and incubated at 37°C in a humidified 5% CO_2_ incubator overnight. Two fold serial dilutions of toxins (100 to 1.56 μg/mL) were added to the cell culture. Cells treated with PBS were used as untreated control. After 48 h incubation at 37°C, DMEM containing MTT at the final concentration of 1 mg/mL was added to each well, incubated for 1 hour and replaced with 100 μl of DMSO to solubilize the formazan crystals. Surviving cells were measured by optical density (OD) at 562 nm, using a spectrophotometer. In order to support the results obtained from MTT assay, cell viability was also analyzed by using Cell Titer-Blue Luminescent Cell Viability Assay (Promega) according to the manufacturers’ protocol. The 50% cytotoxic concentration (CC_50_) was defined as the concentration required to reduce the cell number by 50% compared to that for the untreated control. All experiments were performed in triplicates and repeated a minimum of three times.

### Luciferase-based replication assay

Huh-7.5 cells stably harboring the SGR-Feo-JFH-1 or electroporated with SGR BM45-Feo were seeded into 96-well plates at a density of 5 x 10^3^ per well and toxins at specific concentrations were added. After 48 h, cells were harvested by lysis with Passive Lysis Buffer (Promega, USA) and HCV RNA replication was quantified by measuring luminescence levels using the Luciferase Assay System (Promega, USA). The effective concentration of toxins that inhibit 50% (EC_50_) of replication was calculated using GraphPad Prism software. Cytotoxicity assays were carried out in parallel to determine the CC_50_, using a MTT-based system as described above. The values of CC_50_ and EC_50_ were used to calculate the selectivity index (SI = CC_50_/EC_50_).

### Combined treatment assay

Huh-7.5 cells stably harboring the SGR-Feo-JFH-1 were seeded into 96-well plates at a density of 5 x 10^3^ the day before the assay was carried out. Toxin and Sofosbuvir were added separately, or combined at sub-EC_50_ concentrations. After 48h. cells were harvested by lysis with Passive Lysis Buffer (Promega, USA) and HCV RNA replication was quantified by measuring luminescence levels using the Luciferase Assay System (Promega, USA), as described in Luciferase-based replication assay.

### Virus assays

JFH-1 HCVcc particles [26] were generated as described previously [27]. Huh-7.5 cells were infected with virus at a multiplicity of infection (MOI) of 0.4 and toxins at 10 μg/mL were added at different time points, depending on the stage of HCV life cycle to be evaluated (as described below). To virus titration, HCVcc supernatants were 10-fold serial diluted in DMEM medium and used to infect Huh-7.5 cells. Cells were fixed with 4% paraformaldehyde (PFA) 48 h post-infection (hpi), washed with 100 mM Glicine (Applichem, USA) and semi-permeabilized with 0.1% Triton X-100 (Vetec Labs, BR). Intracellular virus was detected by indirect immunofluorescence using a sheep anti-NS5A IgG [28] as primary antibody and anti-sheep IgG, Alexa Fluor 594 conjugated, as secondary antibody. Infectivity was expressed as focus-forming units per milliliter of supernatant (FFU/mL). All assays were performed in triplicates and repeated a minimum of three times.

### Antiviral activity against HCV replication

Huh-7.5 cells were seeded the day before the assay was carried out. Toxins were diluted to the stated final concentrations in DMEM media. Cells were infected with infectious supernatant for 4 h, washed extensively with PBS to remove non-endocytosed virus particles and toxins were added. After 48 h intracellular virus was titrated. PBS was used as untreated control and cyclosporine A (CsA, Sigma-Aldrich) as control of inhibition of replication [29].

### Inhibitory effects on entry steps

Infectious supernatant containing JFH-1 HCVcc was used to infect naive Huh-7.5 cells in the presence of toxins for 4 h at 37°C. Cells were extensively washed with PBS and replaced with fresh complete medium.

For virucidal assay, infectious supernatant was prior incubated with toxins for 1 h at 37°C and then used to infect naive Huh-7.5 cells. Virus and toxin were incubated with cells for 4 h at 37°C. The inoculum was removed; cells were washed three times with PBS to completely remove virus and toxins, and replaced by fresh media.

For both assays, virus was titrated 48 hpi. PBS and (-)-epigallocatechin gallate (EGCG, Sigma-Aldrich, USA) [30] were used as controls.

### Pre-treatment assay

Huh-7.5 cells were incubated with toxins for 1 hour at 37°C in a humidified 5% prior to infection. After incubation, cells were washed extensively and incubated with HCVcc JFH-1 virus for 4 h. Infectious supernatant was removed, additional washes were performed to completely remove non-endocytosed virus and fresh media was added. Virus was titrated 48 hpi as described above. PBS and EGCG were used as controls.

### Toxins activity on viral release

Huh-7.5 cells infected with HCVcc JFH-1 were seed 48 h prior the treatment. Then, fresh media with toxins at 10 μg/mL was added following a previously described protocol [31]. The plate was gently agitated 24 h post-treatment to mechanically release particles bound to cells and the supernatant was collected, filtered, and stored at—80°C. Intracellular RNA was also extracted by using TRIzol reagent (Life Technologies, Carlsbad, CA, USA) and stored at—80°C. Both procedures followed Nahmias et al. (2008) protocol for HCV secretion analysis.

Extracted RNA was used on cDNA synthesis using High-Capacity cDNA Archieve (Applied Biosystems, Foster City, CA, USA). HCV expression analysis was performed for detection of the HCV5’UTR region using TaqMan Universal PCR Master Mix no AmpErase UNG (Applied Biosystems, Branchburg, NJ, USA). The amplification of the endogenous gene GAPDH was used to normalize levels of expression. PBS was used as negative control and naringenin (NR) at 400 μM was used as positive control of HCV secretion inhibition.

### dsRNA intercalation assay

A dsRNA intercalation assay was performed based on the protocol described by [32]. HCV JFH-1 3’ untranslated region (UTR) (accession no. AB047639) was amplified by a PCR reaction using specific primers flanked by T7 promoter region (forward: 5´TAATACGACTCACTATAGGGGGCACACACTAGGTACA3´; reverse: 5´TAATACGACTCACTATAGGGACATGATCTGCAGAGAG3´; T7 sequences are underlined). The PCR product of 273 bp was purified using the Zymoclean Gel DNA recovery Kit (Zymo Research) and used as template for *in vitro* transcription with the T7 RiboMAX^™^ Express Large Scale RNA Production System kit (Promega). The synthetized dsRNAs were treated with RNase A for 2 h in a 0.3 M NaCl solution and confirmed by RNA denaturation agarose gel (1%) analysis (RNase A does not cleave dsRNA in 0.3 M NaCl solution). To investigate the dsRNA intercalation properties of the compounds, 15 mM dsRNA were incubated with each toxin at 10 μg/mL for 45 m and submitted to agarose gel analysis. PBS and Doxorubicin (DOX) at 400 μg/mL [32] were used as negative and positive control, respectively.

### CD-81 receptors assay

Huh-7.5 cells were seeded to 96-well plate the day before the assay and incubated at 37°C in a humidified 5% CO_2_ incubator. The media was removed, cells were incubated for 30 minutes with CD81/TAPA1 antibody (Thermo Scientific) and the respective toxin at 10 μg/ml in PBS containing 1% BSA (Bovine Serum Albumin—Sigma-Aldrich). After treatment, cells were washed with PBS and incubated with secondary antibody Alexa Fluor 594 (Thermo Fisher). After this, cells were washed with PBS, fixed with 4% paraformadehyde (vol/vol), stained for nuclei with 4 =, 6-diamidino-2-phe-nylindole (DAPI) and analyzed on Fluorescence microscopy ZEN lite 2012.

### Western blot analysis

Cells were lysed in CelLytic^™^ lysis buffer (Sigma-Aldrich) added of protease inhibitors (Sigma-Aldrich). Ten micrograms of protein were resolved by SDS/PAGE and transferred to a PVDF membrane. Membranes were blocked in 10% (w/v) dried skimmed milk powder in Tris-buffered saline with 0.1% Tween-20 (TBS-T). Membranes were probed with anti-NS5A IgG (Macdonald et al., 2003) or mouse anti-GAPDH IgG (AbCam) in 5% (w/v) dried skimmed milk in TBS-T. The antibodies were detected with the secondary horseradish peroxidase-conjugated antibody and in-house enhanced chemiluminescent reagent.

### Immunofluorescence assay

Cells grown on glass coverslips were fixed for 10 min with 4% (vol/vol) paraformaldehyde in PBS at room temperature, washed with 100 mM Glicine (Applichem, USA) and semi-permeabilized with 0.1% Triton X-100 (Vetec Labs, BR). Cells were washed with PBS and blocked in PBS–1% bovine serum albumin (BSA) for 30 min prior to incubation with primary antibodies for 1 h in PBS–1% BSA with polyclonal sheep anti-NS5A IgG [27] as primary antibody and anti-sheep IgG, Alexa Fluor 594 conjugated, as secondary antibody. Cells were washed, mounted onto microscope slides and labelled for nuclei and lipid droplets (LDs) labels with DAPi and BODYPI 493/503, respectively.

### Statistical analysis

Differences between means of readings were compared using analysis of variance (one-way or two-way ANOVA) or Student *t* test using GraphPad Prism 5.0 software (GraphPad Software). *P* values of less than 0.001 (indicated by asterisks) were considered to be statistically significant.

## Results

### Inhibitory effect of toxins on HCV replication

**Fig 2:**
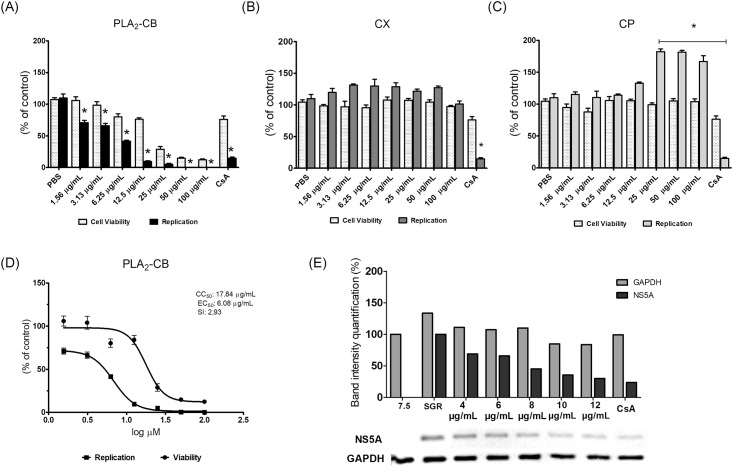
Inhibitory activity of the toxins on HCV replication. Huh-7.5 cell line stably expressing SGR-luc-JFH-1 were treated with PLA_2_-CB (A), CX (B), CP (C) at specific concentrations for 48 h. The effective concentration of inhibition (EC_50_), the cytotoxic concentration of 50% (CC_50_), and the selectivity index (SI = CC_50_/EC_50_) were calculated (D). Expression of HCV NS5A protein was measured 48 h post-treatment using western blotting assays (E). Mean values of three independent experiments each measured in triplicate including the standard deviation are shown. P < 0.001 was considered significant (*).

To evaluate the potential effect of the CX, CP and PLA_2_-CB toxins on HCV replication, we first used a subgenomic replicon system. Huh-7.5 cell line stably expressing SGR-FEO-JFH-1 (Huh-7.5-SGR-FEO-JFH-1) were treated with two fold serial dilutions (100–1.56 μg/mL) of each toxin for 48 h to assess the effect of these compounds on both HCV replication and cell viability (Fig 2). The results showed that PLA_2_-CB significantly (p< 0.001) inhibited HCV replication at non-cytotoxic concentrations (Fig 2A); while CX did not demonstrate any effect on SGR replication (Fig 2B). In contrast, cells treated with 100, 50 and 25 μg/mL of CP significantly (p< 0.001) increased HCV replication rates (Fig 2C). Treatment of cells with PLA_2_-CB decreased HCV replication in a dose-dependent manner with EC_50_ of 6.08 μg/mL, CC_50_ of 17.84 μg/mL and a SI of 2.93 (Fig 2D). As shown in Fig 2E, expression of HCV NS5A protein was also gradually reduced in the presence of increasing non-cytotoxic doses of PLA_2_-CB (20–4 μg/mL). To support MTT data, cell viability was also analyzed by using Cell Titer-Blue Luminescent Cell Viability Assay (Promega) (S1 Fig). No significant differences were observed in CX and CP cell viability. However, a significant difference was observed for PLA_2_-CB at 100, 50 and 25 μg/mL concentrations, showing an increase in viability when compared to the MTT assay.

**Fig 3:**
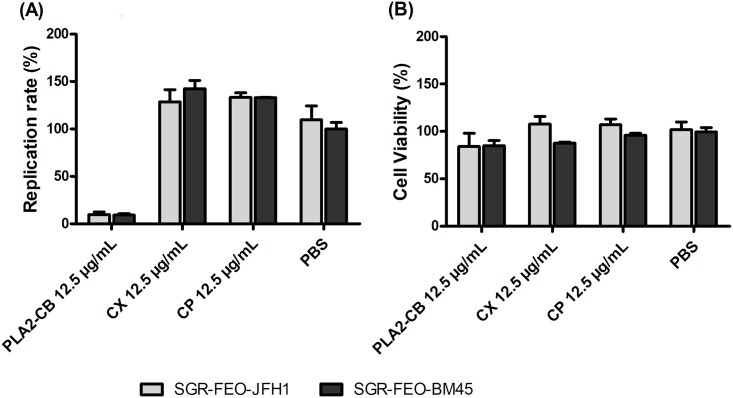
Effect of toxins on genotype 1b. Huh-7.5 cells were electroporated with 2 μg of RNA SGR-Feo-BM-45 genotype 1b and seeded into 96-well plate. After 24 h, cells were treated with PLA_2_-CB, CX or CP at 12.5 μg/mL and maintained for 48 h. HCV replication was quantified by measuring luminescence levels (A) and cell viability absorbance (B). P < 0.001 was considered significant (*).

The antiviral activity of PLA_2_-CB was also evaluated against the replication of HCV genotype 1b by using a subgenomic system (genotype 1b SGR BM45-Feo). Results corroborated the data observed for genotype 2a (Fig 3A and 3B), no significant differences were observed between the two systems. Subsequent assays were performed with toxins at 10 μg/mL final concentration (favorable ratio of cytotoxicity to antiviral potency).

**Fig 4:**
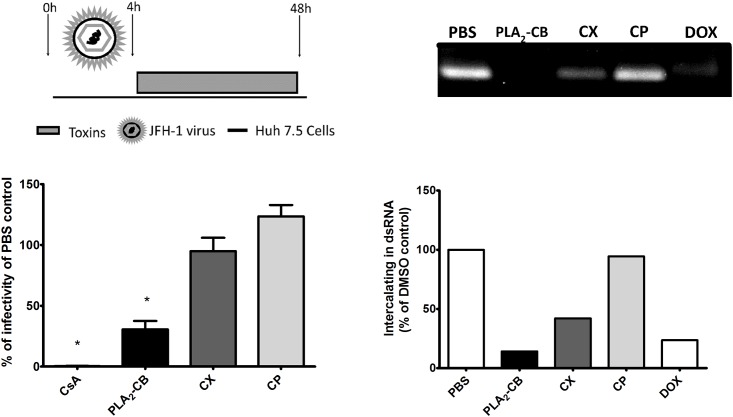
Effect of toxins on HCV replication. Huh7.5 cells were infected with JFH-1 HCVcc for 4 h, Then cells were washed extensively to remove virus and treated with toxins. Replication levels were assessed by performing the focus formation units assay 48 h post-infection. PBS was used as negative control and CsA as positive control for replication inhibition. Mean values of three independent experiments each measured in triplicate including the standard deviation are shown. P < 0.001 was considered significant (A). In an attempt to determine de antiviral mechanism of action of these toxins, synthesized dsRNA was incubated with each toxin for 45 min and submitted to electrophoresis in 1% agarose gel. PBS was used as negative control and Doxorubicin (DOX) was used as intercalating positive control (B).

We next analyzed the effects of the toxins CX, CP and PLA_2_-CB on genome replication in the context of full length virus. Huh-7.5 cells were infected with JFH-1 HCVcc at MOI 0.4 for 4 h, washed to remove non-endocytosed virus particle and added of toxins. Replication levels were assessed by the focus formation units assay 48 h post-infection. Consistent with the SGR data, PLA_2_-CB effectively blocked (p< 0.001) virus replication (Fig 4A). No significant differences on virus replication rates were observed by treating infected cells with CX or CP (p< 0.001). These results suggest that only the isolated form of PLA_2_-CB is able to reduce HCV replication, whereas it has no effect on replication when associated with CP to form the CX complex.

In an attempt to elucidate the mechanism in which PLA_2_-CB inhibits HCV replication, we performed a standard methodology to analyze the potential of the toxins to intercalate into dsRNA. As shown in Fig 4B, treatment with PLA_2_-CB reduced 86% of dsRNA stained by ethidium bromide (Fig 4B). CX showed an intermediate reduction (58%); while CP demonstrated no effect on the dsRNA. The positive control doxorubicin decreased 77% of dsRNA stained and demonstrated to be less efficient than PLA_2_-CB (Fig 4B). These results suggest that the inhibition of replication by PLA_2_-CB may be due to its dsRNA intercalation properties.

**Fig 5:**
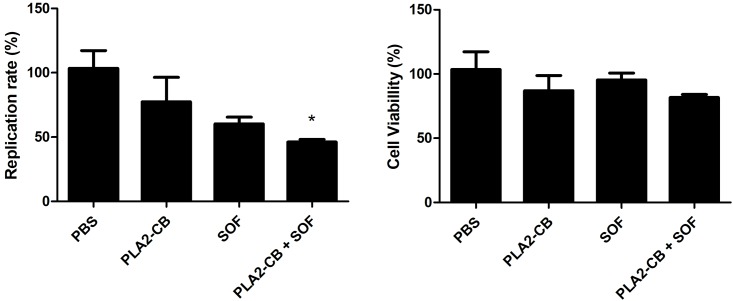
Sofosbuvir and PLA_2_-CB combined treatment. Huh-7.5 cells stably harboring the SGR-Feo-JFH-1 were seeded into 96-well plates and treated with PLA_2_-CB, Sofosbuvir or the combination PLA_2_-CB and Sofosbuvir for 48 h. After the treatment replication level (A) and cell viability (B) were measured. P < 0.001 was considered significant (*).

We also evaluated the effect of PLA_2_-CB in the presence or absence of the DAA Sofosbuvir [33] at sub-EC_50_ concentrations. Huh-7.5 cells stably harboring the SGR-Feo-JFH-1 were seeded into 96-well plates and treated with the respective compound after 48 h the luciferase levels were measured. PLA_2_-CB and Sofosbuvir were diluted separately in DMEM, to be incubated at the final concentrations 3,8 μg/mL and 150 ɳM, respectively, in monotherapy or in combined treatment. At the sub-EC_50_ concentrations, PLA_2_-CB or Sofosbuvir inhibited up to 22.7% and 39,7% of HCV replication, respectively, and combined therapy significantly increased the replication blockage up to 55.8% (p< 0.001) (Fig 5A) at non-cytotoxic concentrations (Fig 5B).

### Crotoxin complex and its subunit PLA_2_-CB inhibited HCV entry

**Fig 6:**
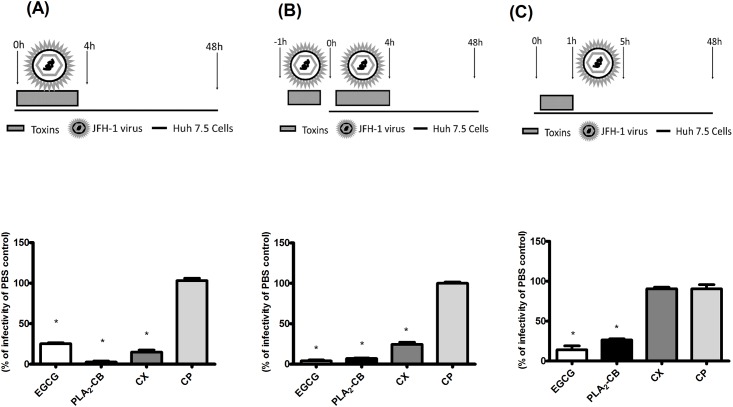
Effect of the toxins on HCV infectivity. Infectious supernatant and toxins were added in different times to the cells and intracellular virus was titrated 48 h post-infection by analyzing focus-forming units per milliliters (Ffu/mL). For entry assay, Huh-7.5 cells were infected with JFH-1 HCVcc and toxins were immediately added. After 4 h, the supernatant was replaced by fresh medium after repeated washes with PBS to remove completely the inoculum (A). For virucidal assay, JFH-1 HCVcc particles were incubated with toxins for 1 h prior to the infection. After that, the inoculum was used to infect naïve Huh-7.5 cells for 4 h. Cells were extensively washed and medium was added (B). In the pre-treatment assay, cells were previously treated with toxins for 1 h, washed to completely remove toxins and infected with JFH-1 virus for 4 h. Cells were then washed to virus removal and replaced with fresh media for up to 48 h post-infection (C). PBS was used as negative control and EGCG as control of entry blockage. Mean values of three independent experiments each measured in triplicate including the standard deviation are shown. P < 0.001 was considered significant.

The effect of the toxins CX, CP and PLA_2_-CB on different stages of HCV entry was investigated. First, Huh-7.5 cells were infected with JFH-1 HCVcc and toxins were immediately added. Cells were incubated with virus plus toxin inoculums for 4 h, washed to completely remove the inoculum and replaced with fresh medium for 48 h. Intracellular virus were titrated by using focus formation units assay. The results demonstrated that CX and PLA_2_-CB were able to block 85% and 97.3% of HCV entry, respectively. Contradictory, CP showed no inhibitory effect on viral entry (Fig 6A). It suggests that the inhibition of HCV entry might be related to the catalytic action of PLA_2_-CB. The activity observed in CX treatment may be due to the PLA_2_-CB presence into the CX complex.

To characterize the antiviral effect of CX and PLA_2_-CB on virus entry, we next assessed the impact of these toxins on the HCV viral particle by performing a virucidal assay. JFH-1 HCVcc supernatant was incubated with each toxin in cell-free conditions for 1 h prior to the cells infection. The inoculum was then used to infect Huh-7.5 cells for 4 h. Cells were washed with PBS to remove the inoculum and replaced with fresh media. CX and PLA_2_-CB demonstrated a significant virucidal activity, blocking 75.5% and 93% of virus entry, respectively (p<0.001) (Fig 6B). CP also demonstrated no virucidal activity on HCV virus. This result could suggest that the antiviral effect of CX and PLA_2_-CB observed was due to a direct action of these toxins on the virus particle structure.

We further investigated whether the blockage of viral entry by the toxins is influenced by its activity on the host cells. Huh-7.5 cells were previously treated with toxins for 1 h and then infected with JFH-1 HCVcc virus for 4 h. Intracellular virus was titrated 48 hpi. The analysis showed that treatment with PLA_2_-CB prior to infection significantly inhibited 73.5% of HCV infectivity (Fig 6C). Therefore, PLA_2_-CB may also act somehow on the host cells to decrease infectivity. In contrast, previous treatment on cells with CX and CP had not effect on HCV infectivity (Fig 5C), suggesting that PLA_2_-CB probably lacks the antiviral activity when associated with CP to form CX.

**Fig 7:**
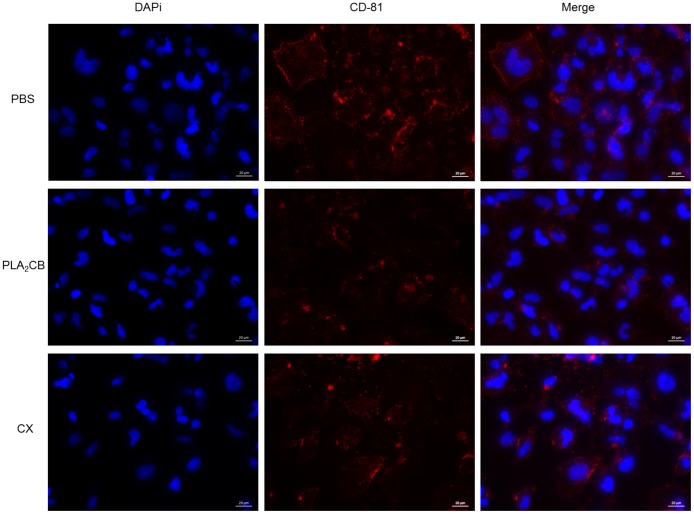
Effect of toxins on CD81 cell receptors. Huh-7.5 cells were incubated with CD81/TAPA1 antibody and 10 μg/ml of CX, PLA_2_-CB or PBS. Then cells were washed and incubated with secondary antibody Alexa Fluor 594. Cells were fixed with 4% paraformaldehyde and labelled for nuclei with DAPI and analyzed on Fluorescence microscopy ZEN lite 2012.

We also analyzed whether the antiviral activity of PLA_2_-CB or CX is due to an interaction between these toxins and CD-81 cell receptors. However, our data suggested that the antiviral activity of toxins is not by binding to CD-81 cell receptors (Fig 7).

### HCV release is inhibited by crotapotin and crotoxin but not by PLA2-CB

**Fig 8:**
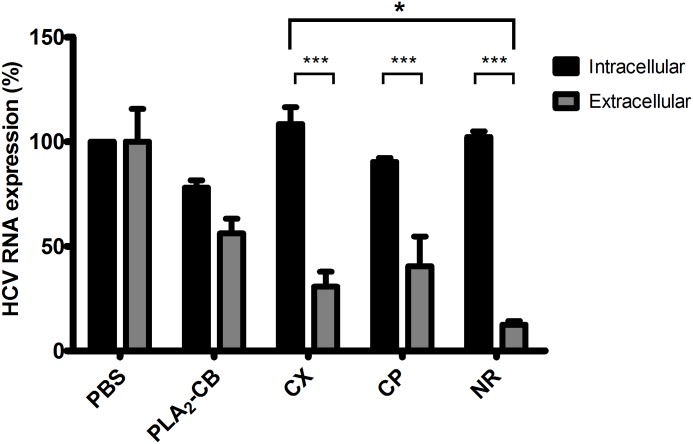
Antiviral activity of toxins on HCV release. Huh-7.5 cells previously infected with JFH-1 virus was plated 48 h prior treatment. The toxins were added at 10 μg/mL and incubated for 24 h. Supernatant was collected and cells were harvested, and intra and extracellular RNA were quantified by qPCR. PBS was used as negative control and naringenin (NR) at 400 μM was used as positive control of HCV secretion. Mean values of three independent experiments each measured in triplicate including the standard deviation are shown. P < 0.001 was considered significant.

Finally, we investigated the effect of toxins on HCV release. HCVcc JFH-1 infected cells were treated with each toxin at 10 μg/mL for 24 h. Supernatants were collected and cells were harvested, and viral RNA was quantified by qPCR. Intra and extracellular virus tires were analyzed in order to evaluate the amounts of virus produced (intracellular) and released (extracellular). Treatment with PLA_2_-CB showed no significant difference between intra and extracellular virus demonstrating no effect on virus release—all virus produced is released (PLA_2_-CB affects earlier steps of HCV life cycle, then lower amounts of virus is produced but is also released) (Fig 8). The reduction of both intra and extracellular viral RNA observed for PLA_2_-CB compared to the PBS control was the consequence of its effect on HCV replication. In contrast, the treatment with CX and CP demonstrated significant difference on levels of intra and extracellular HCV RNA and therefore, on virus release (virus is produced in earlier steps but not released from cells). The results showed that CX and CP reduced 50 and 78% of HCV release, respectively (Fig 8). Interestingly, the interpretation of these results may suggest that the inhibitory action of CX on HCV release, could be related to the presence of subunit CP in this complex, and its effect it is not influenced by the interaction of subunits to form CX complex.

### PLA_2_-CB and crotapotin affect lipid metabolism in Huh-7.5 cells

**Fig 9:**
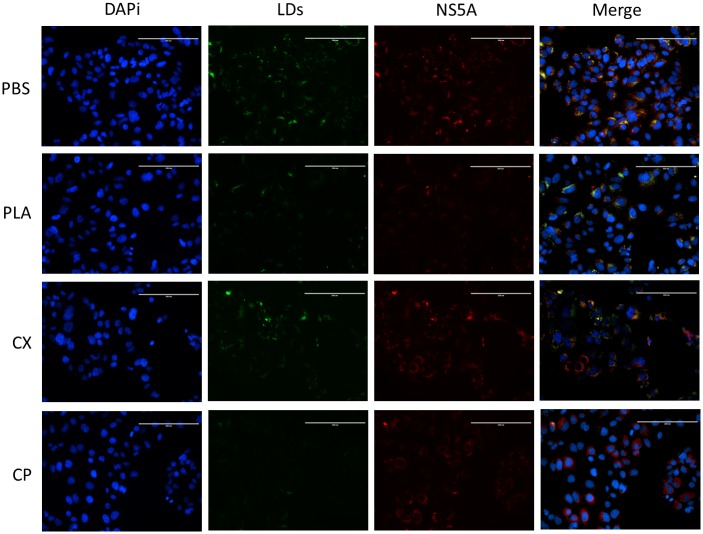
Effect of toxins on lipids droplets. Huh-7.5 cell line stably expressing SGR-luc-JFH-1 were treated with 10 μg/mL of CX, CP or PLA_2_-CB at 37° for 48 hours. Cell were fixed and nuclei, Lipid droplets (LDs) and viral protein NS5A were labelled with DAPi (blue), BODYPI 493/503 (green) and antibodies against NS5A (red), respectively. PBS was used as untreated control. Scale bars, 200 nm.

Given the described links between the HCV life cycle and the host lipid metabolism, we evaluated whether the treatment with toxins disturbed the lipid metabolism of Huh-7.5 cells stably harboring the SGR-Feo-JFH-1. The treatment with PLA2-CB or CP at 10 μg/mL decreased of LDs levels compared to untreated control, but no effect was observed for CX (Fig 9). As expected, PLA_2_-CB also reduced NS5A protein levels due to the antiviral activity on HCV replication as shown in Figs 2, 3 and 4. Therefore, PLA_2_-CB reduced both viral protein LDs levels. Interesting, CP also interferes in lipid metabolism but did not reduced HCV replication levels levels, in accordance to the data presented on Figs 2, 3 and 4. However, since HCV assembly occurs in close proximity to lipid droplets [34,35], the reduction of LDs by CP could be related to its antiviral effect on HCV release (Fig 8).

## Discussion

The current available therapy for Hepatitis C is not effective for all treated patients, is limited by side effects and is expensive. Therefore, there is an evident need to develop new therapeutic approaches that result in optimal response rates, milder side effects and lower cost of production.

Several studies have described compounds isolated from snake venoms with anti-bacterial, anti-inflammatory, anticancer and antiviral activity [17,36]. Since the discovery of captopril, a potent anti-hypertensive drug based on snake venom protein [37], many toxins are being investigated due to their therapeutic potential. Toxins isolated from *Crotalus durissus terrificus* were previously screened for their activity against other viruses of *Flaviviridae* family and demonstrated to be effective on blocking the early steps of the life cycle of these viruses [19]. CX was initially described with neurotoxic and myotoxic activity however over the years new activities like anti-inflammatory, antimicrobial and antitumor have been described for this complex [38,39].

In this study, we investigated the antiviral activities of the heterodimeric complex CX, and its subunits CP and PLA_2_-CB, isolated from *Crotalus durissus terrificus* venom against HCV infection *in vitro*. We were able to demonstrate the multiple antiviral effects of these toxins which inhibited different stages of the HCV life cycle.

Crotoxin is a noncovalent association formed by two subunits, acidic subunit CP (9.5 kDa) formed by three polypeptide chains (α, β, and γ) cross-linked by seven disulfide bonds and basic subunit PLA_2_-CB (14.5 kDa). Subunit B is a phospholipase formed by a single chain of 122 amino acid residues cross-linked by seven disulfide bonds [40,41].

Our data showed that PLA_2_-CB blocked viral entry by both the action on the virus particle and somehow on the host cells. It is consistent with findings of a previous study which demonstrated that the PLA_2_-CB inhibits the early stages of replication cycle of two members of the Flaviviridae family, DENV and YFV [19]. The authors showed that PLA_2_-CB reduced virus infection by acting on the host cells and/or on the viral particles. Additionally, Rojas and collaborators demonstrated that the incubation of quercetin with HCVcc in a free-cell condition prior to the infection of cells significantly reduced HCV infectivity. By their results, the authors suggested that quercetin acts directly on HCV, modifying the integrity of viral particles [42]. Therefore, our analyses are in agreement with previous data and could suggest that the antiviral effect of CX and PLA_2_-CB observed was due to a direct action of these toxins on the virus particle structure. However, we acknowledge that interpretation of these experiments is challenging and we therefore cannot rule out the possibility that these compounds possess an independent effect on the host cell components of the receptor complex or interacts with components which modulate viral entry. We investigated whether the toxins could abrogate virus entry by interacting with CD-81 cell receptor, an important factor for HCV infection [43,44]. Our results suggest that the antiviral activity of toxins CX and PLA_2_-CB on HCV entry is not by blocking CD-81 cell receptors.

PLA_2_-CB is a phospholipase which belongs to the group II of secreted enzymes that hydrolyze glycerophospholipids at the sn-2 position, producing lysophospholipids and fatty acids [45]. They represent a versatile class of enzymes which play a key role in various biological activities, such as lipid digestion, host defense and homeostasis of cellular membranes [45,46]. Once lipids are considered an essential part and associated with different steps in HCV life cycle [47,48], we evaluated the effect of the toxins on LDs. The results demonstrated that PLA_2_-CB was able to decrease the amount of LDs in Huh-7.5 cells combined to the reduction of NS5A protein levels and blockage of HCV replication. Previous studies also showed that alterations in LDs are essential to HCV replication [49], and LDs also seem to be extremely associated to virus replication complex [3,50].

Rojas et. al. [42] also showed that genes involved in lipid synthesis and uptake (Low-density lipoprotein receptor (LDLr), fatty acid synthesis (FASN), acetyl-CoA carboxylase (ACC), and transcription factor binding of sterol regulatory elements 1 (SREBP1c) significantly increased. The expression of the microsomal enzyme diacylglycerol acyltransferase (DGAT), related to the synthesis of triglycerides and interaction with the core protein for the formation of infectious particles, also showed a significant increase. The increase of DGAT, ACC, LDLr e SREBP1c, LDLr and SREBP1c expression were completely inhibited by treatment with quercetin at 50 uM. The effect of quercetin on the morphology of lipid vesicles was also evaluated, and a morphometric analysis showed a reduction in the radius, area and volume of lipid droplets [42].

Nordihydroguaiaretic acid (NDGA) derived from the species *Larrea tridentata* inhibited 40% HCV virus replication in 48 hours of treatment when compared to untreated controls. In addition, NDGA altered lipid metabolism by preventing activation of the SREBP1c gene, LDLr and FASN target receptors in Huh-7 infected with HCV. The authors also observed that NDGA treatment induced lipid vesicle rearrangement, decreasing in number and increasing size. When Huh-7 cells were stimulated with oleic acid and infected with HCV, treatment with NDGA reduced the number and size of lipid vesicles, this can be explained by vesicle fusion or secretion of pre-synthesized vesicles [51].

In view of this we suppose that the PLA, CX and CP compounds are inhibiting the expression of genes responsible for lipid metabolism (DGAT, ACC, LDLr and SREBP1c), which makes it difficult to assemble the viral particles and thus inhibit replication.

More recently, the effect of PLA_2_-CB against DENV was associated to its catalytic activity, inactivating virus particle probably by cleavage of glycerophospholipid of the virus envelope [52]. These results were also described for other enveloped virus such as *Rocio virus*, *Oropouche virus*, *Mayaro virus* [16] and *Human Immunodeficiency Virus* (HIV) [15]. Since HCV is an enveloped virus [53] and viral particles are also associated with lipoproteins [54], it is plausible to suggest that the virucidal effect observed in our analysis could be due to the phospholipase activity attributed to PLA_2_-CB, however, more studies are necessary to confirm this possibility.

Our results demonstrated that PLA_2_-CB reduced HCV replication in either subgenomic reporters SGR-Feo-JFH1 (genotype 2a) or SGR-BM45-Feo (genotype 1b), and also by using the full-length JFH1 systems. When the inhibitory effects were further investigated, we found that PLA_2_-CB strongly intercalated into dsRNA, suggesting a possible mechanism of action in which PLA_2_-CB inhibits HCV replication. The association of PLA_2_-CB with CP decreased the intercalation property, consistent with the results observed for CX and CP. This result also could explain the discrete but not significant reduction of full length virus replication by CX when compared to CP treatment. We believe that either the use of two different systems could explain the differences between the results or that the effects of CX in the dsRNA is not strong enough to reduce replication levels, since the dsRNA is produced. The capacity of intercalate into dsRNA was also previously described for other natural compounds as amidinoanthracyclines [32] and acridones [55] against HCV. PLA_2_-CB also showed a slight increase in replication blockade when combined with Sofosbuvir. This finding added to the action of the PLA_2_-CB in other steps could provide complementary mechanisms of action to HCV inhibition. [56]Despite CX did not significantly inhibit HCV replication, this complex was effective in blocking the HCV entry and release stages. The observed data suggests that the effect on HCV entry is mainly due to its virucidal activity. CX is characterized as a heterodimeric protein complex consisting of two subunits, an acidic component (CP) and a basic subunit (PLA_2_-CB) [21,57–59]. Therefore, the virucidal effect of CX is probably due to the presence of PLA_2_-CB in this complex. Altogether, our data shows a stronger virucidal effect when cells are treated with the isolated form of PLA_2_-CB, and a significant but reduced effect when it is in the complex form. This pattern of activity was similarly found concerning other biological activities of the CX. According to Sampaio *et al*., the enzymatic activity related to CX was also associated with the presence of the subunit PLA_2_-CB [39].

Interestingly, our data demonstrated that CP possesses inhibitory effect on HCV release and interferes with lipid metabolism, but did not reduced NS5A protein levels. CP was initially described with no catalytical or cytotoxic activities and acts mainly preventing PLA_2_-CB to perform unspecific interactions [60–62]. Miyanari, *et al*. demonstrated that some steps of virus assembly occurs around LDs [34]. Additionally, the flavonoid Naringenin demonstrated to inhibit the secretion of HCV particles, without affecting intracellular levels of viral RNA or protein [63]. This finding corroborated with our data, therefore we hypothesize that the antiviral effect of CP on HCV release could be due to the decrease of LDs in treated Huh-7.5 cells.

## Conclusion

In summary, we demonstrated that toxins isolated from *Crotalus durissus terrificus* can inhibit different stages of HCV life cycle as entry, replication and release. The mechanisms of antiviral action were: (a) block HCV entry by the direct action of PLA_2_-CB on the viral particle and/or interference on the host cells; (b) reduce replication possible by intercalating to the intermediate dsRNA formed during the replication process; (c) disrupt replication or release by decreasing levels of LDs and interferes with host lipid metabolism. These results may be useful for the development of future therapies and for a better understand of how these toxins act inhibiting virus machinery.

## Supporting information

S1 Fig Cell viability by Cell Titer-Blue kit. (TIF)
