# Supplementary figures and images for: Multiple effects of toxins isolated from Crotalus durissus terrificus on the hepatitis C virus life cycle
Source: PLoS One. 2017 Nov 15;12(11):e0187857. doi: 10.1371/journal.pone.0187857 (PMC5687739; doi:10.1371/journal.pone.0187857)

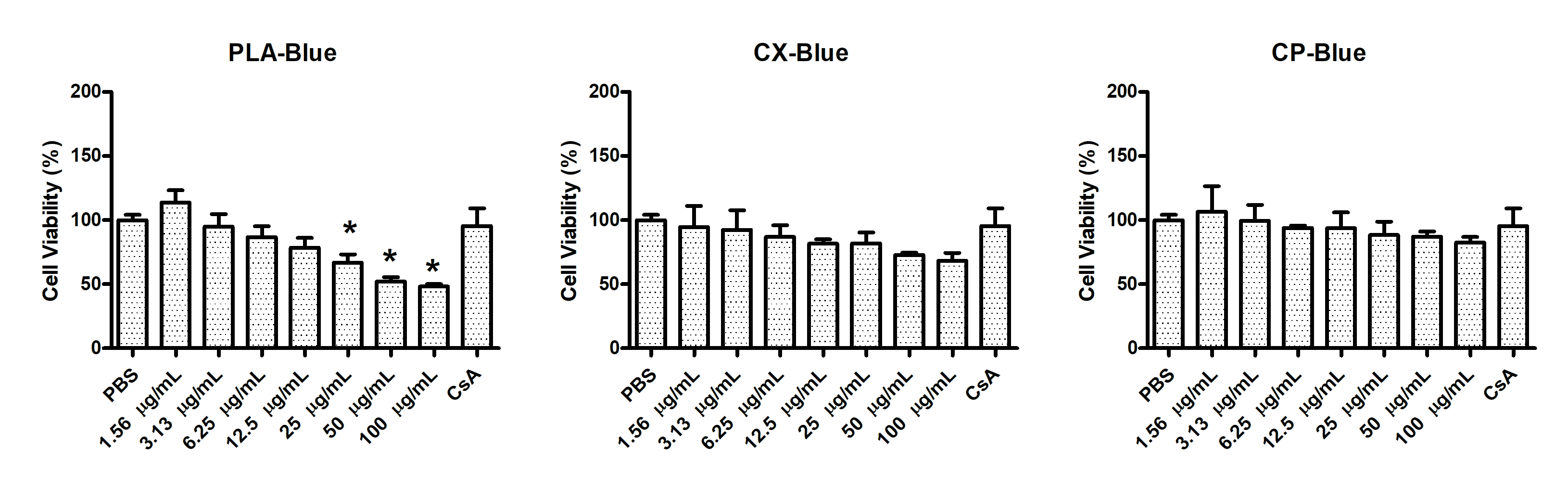

Supplement: S1 Fig — (TIF) [file pone.0187857.s001.tif]
